# Engagement present and future: Graduate student and faculty perceptions of social media and the role of the public in science engagement

**DOI:** 10.1371/journal.pone.0216274

**Published:** 2019-05-02

**Authors:** Emily L. Howell, Julia Nepper, Dominique Brossard, Michael A. Xenos, Dietram A. Scheufele

**Affiliations:** 1 Nelson Institute for Environmental Studies, University of Wisconsin-Madison, Madison, Wisconsin, United States of America; 2 Promega Corporation, Fitchburg, Wisconsin, United States of America; 3 Department of Life Science Communication, University of Wisconsin-Madison, Madison, Wisconsin, United States of America; 4 Morgridge Institute for Research, Madison, Wisconsin, United States of America; 5 Department of Communication Arts, University of Wisconsin-Madison, Madison, Wisconsin, United States of America; Mayo Clinic, UNITED STATES

## Abstract

Interest in public engagement with science activities has grown in recent decades, especially engagement through social media and among graduate students. Research on scientists’ views of engagement, particularly two-way engagement and engagement through social media, is sparse, particularly research examining graduate students’ views. We compare graduate students and faculty in biological and physical sciences at a land-grant, research-intensive university in their views on engagement. We find that both groups overwhelmingly believe that public input in decision-making around science issues is important, and hold largely pro-engagement attitudes. Graduate students, however, have somewhat more optimistic views of engagement through social media and on the appropriateness of discussing science controversy on social media. We discuss implications for graduate education and future engagement.

## Introduction

Since the 1990s, U.S. academic research funders and institutions have placed greater importance on incorporating public engagement in scientific research [1]. With the growth of community- and engagement-focused research and outreach by academics from U.S. universities, some researchers have called this period of increased engagement a “renaissance… in higher education” [1]. The shift toward greater engagement, however, has been slow. Additionally, its current and future progress depends on institutional structures that support engagement and on changing norms and culture among graduate students, faculty, and administrators toward valuing engagement as part of academic careers [1–3]. Evidence exists that norms are changing, however, especially among graduate students who appear to have a strong and possibly growing interest in making engagement an integral, or at least important, part of their academic careers [2, 4, 5].

In addition to the academic environment, several changes to the media environment in which engagement occurs have promise for strengthening current and future levels of engagement. The rise of social media and online news and subsequent shrinking of traditional media outlets for science news means online sources are now many Americans’ primary sources for science information [6–8]. The changing media environment offers new outlets for traditional engagement through interviews with journalists and one-directional communication of science, similar to what newspapers and television offer. It also offers opportunities for this kind of communication in ways that require less time and resources and that allow scientists to create their own material through blogs and videos [9]. These changes with online media also create opportunities for scientists to engage in two-directional communication of their research, and communicate at different stages of the research process [10]. This is especially true with the growth of social media, in which information is almost always situated in a greater discourse and context created through users’ comments, likes, hashtags, and related posts.

Unfortunately, little research exists on graduate students’ levels and perceptions of public engagement (for exceptions, see [4, 5]), as well as on scientists’ views of using social media for engagement, although the body of research for the latter has been growing (e.g. [11, 12]). Because of this, we do not know if the apparent increase in graduate student interest in engagement is different from faculty’s views of engagement and in what ways, particularly in the scientific disciplines. We also do not have a sense of the role social media might play in shaping this potential cohort shift between graduate students’ and faculty members’ views of engagement in science fields.

Several factors point to the potential for such a shift, especially through social media. Younger people use social media more frequently and as a larger portion of their overall media diet [13]. Given the opportunities social media can provide for engagement, it is possible that graduate students are more likely to engage through social media. If this is the case, features of engagement on social media could in turn shape the types of engagement that graduate students participate in (whether one- or two-directional communication or in the types of content and issues discussed) as well as graduate students’ views of the public’s role in engagement.

Here we compare graduate students and faculty members in biological and physical sciences at a large, land-grant, research university–examining their engagement levels and views of engagement. With the changing engagement opportunities with social media and the potential for growing prevalence of two-way engagement motivations, we are especially interested in if there are differences between graduate students and faculty members in their views of using social media for engagement and of the roles of scientists and the public when it comes to engaging in science issues. We find that although faculty are significantly more likely to frequently engage with the public and with reporters than are graduate students–consistent with findings that seniority is an important predictor of engagement [14–17]–graduate students are less likely to view using social media as too time-consuming. The groups are similar in their positive views of the public [18–20].

Perhaps pointing to potential generational differences, however, we find differences in views of what type of engagement each group sees as appropriate through social media. In particular, graduate students are significantly more likely to believe it is appropriate to discuss controversial science issues in public on social media. We discuss the implications of these views for public engagement as well as the role of institutional factors, which will shape whether these differences capture a lasting shift in views of engagement or whether they are merely reflecting graduate students’ views before they are socialized to share the perspectives of the faculty they are training to become.

## Background

### History of public engagement with science in the U.S.

Interest in public engagement has grown as people increasingly perceive science as integrated with and expected to respond to political issues and societal values [21]. These expectations are due to a mixture of factors including increased federal funding of science, scientific developments with large implications for society, and greater push from institutions, researchers, and the public for discourse on policy-relevant scientific issues. Although science has always been connected to politics and society, these connections became more explicit when the U.S. government reinforced science as a national priority at the end of World War II and start of the Cold War, with the establishment of the National Science Foundation in 1950, dedicated to supporting basic research [21–23]. As science became explicitly a national priority, funded by taxpayers, it required public support as well. Early outreach emerged from the belief that educating the public on science would make them more supportive of science [24]. Science as a national priority also led to goals of developing greater science education and outreach to “recruit” and “cultivate” the next great scientific minds [22].

Although those approaches to public communication of science were often more one-directional, with scientists giving information to the public [25], calls for increased two-directional communication that allows public values to more directly shape scientific research became more common in the latter part of the 20^th^ century. Such calls focused on two-directional communication as a way to ensure public acceptability, achieve democratic goals, and create more objective outcomes through incorporating a wider range of values, perspectives, goals, and concerns [26–29]. The rationale for two-way engagement has strengthened in recent decades as science is increasingly leading to developments, particularly in the bio- and information sciences, that involve great implications for society (see, for examples [10, 30]).

#### NSF and institutionalizing engagement

These changes in perceptions of the relationships between science, society, and the public in the U.S. are mirrored with the National Science Foundation’s (NSF) increased focus on the “broader impact,” or societal gains, of research for evaluating grant proposals. This focus began in 1997 when the NSF established “intellectual merit” and “broader impacts” as the two criterion for assessing grant proposals [31]. Intellectual merit is the potential of the project to “advance knowledge,” and the broader impacts are “the potential to benefit society” [31, 32] Since the 1997 revision, the NSF has also added emphasis to the importance of broader impacts of research by requiring a separate section of applicants’ proposals that specifically addresses the potential for greater societal benefits through the proposed research [33]. As funder of approximately one-fourth of all federally-supported U.S. research in academic institutions and the dominant funding source for several disciplines [34], the NSF’s increased focus on broader impacts means a large number of researchers must participate in public engagement opportunities as a way to meet those requirements.

#### Changing engagement opportunities in the 21^st^ century

Recognizing the increased societal and institutional focus on engagement and a lack of research on what scientists think of engagement, the past two decades have also had an increase in qualitative and quantitative research examining scientists’ views of engagement, as well as discussion defining what it means to “engage”. Literature often uses “engagement” as a catchall for information sharing with the public: from more “two-way” discussions in which researchers both provide and receive information from publics to more “one-way” dissemination of information to or from publics (for examples, see [18, 25, 35]). Similarly, both communication and outreach can often refer to either one- or two-way information sharing [18, 36]. In work that defined engagement by the direction of information sharing involved, Rowe & Frewer [25] specify that “communication” is one-directional information sharing from the organizers of an engagement event to the public, while “consultation” is one-way sharing in the other direction, and public “participation” is two-directional sharing, with all three of these encompassing “engagement”. Here, we do not distinguish between these types of engagement other than to specify when we are interested in more two-way engagement (or Rowe & Frewer’s “participation” [25]). This choice is to reflect the broad focus on this piece and of the survey items we use. We use the vernacular definition of “engagement” to represent interacting with publics, whether in one-way or two-way information sharing, and specify when we are particularly focused on more active, two-way information sharing. Similarly, we use “communication” to refer to any type of information sharing, rather than just unidirectional sharing, reflecting how the term appears in communication research literature (for an example in engagement and communication research, see [37]).

Studies typically focus on what predicts scientists’ likelihood of engaging in outreach activities, how they view the public, and what they perceive as their objectives for outreach (see, for examples, [14, 38]). The body of research is still small, however, and two changes in particular in the 21^st^ century highlight the need for greater research on scientists’ engagement, especially on graduate students’ views of engagement. First, the increased calls for engagement over the past few decades appear to have resulted in more and more graduate students supplementing their academic experience with outreach or choosing to study societal and engagement issues in their research [4, 5]. There is little empirical research, however, on whether graduate students’ engagement behavior or how they envision engagement (views of their role, the role of the public, and types of appropriate engagement and public discourse) is different from researchers at the faculty level.

Second, changes to the media environment with the rise of social media provide new opportunities for researchers to engage and could change what engagement looks like for younger researchers. Although the use of social media in the U.S. is now widespread, younger Americans are still most likely of any age group to use social media, especially in place of more traditional media sources, for entertainment, news, and communication [7]. Shrinking space for science news in traditional television and print journalism also means that a majority of Americans rely on the internet for information on science topics [6]. Although some evidence suggests that younger scientists rely on online information and social networks more than do older researchers [12, 39], no research, to our knowledge, focuses on how graduate students’ use of social media for engagement compares to that of faculty members,

### Factors predicting engagement

Based on the literature that does examine scientists’ views of engagement, we focus in this analysis on three broad categories of factors that can shape frequency and views of engagement: status, perceptions of possible rewards (or of negative outcomes), and views of the public and of the role of the public and scientists in engagement.

#### Status

One of the consistently strongest predictors of frequency of engagement among scientists is the status of a scientist, or where he or she is in his or her career, with high-status, tenured faculty more likely to engage [14–16, 40]. In some cases, such as talking with reporters, this can be due to reporters being more likely to hear about and reach out to more established academics [14, 15]. In cases of direct public outreach, status could also play a role if faculty at the beginning of their careers believe that research or teaching is a bigger priority for advancing their academic career and securing tenure than engagement is [3]. Here we examine status from the view of differences in frequency of engagement of graduate students compared to faculty. Although graduate students do not yet have to worry about the tenure-process, we expect they are less likely to be contacted by reporters than are faculty. They also might be less likely to directly engage with the public either because of fewer perceived opportunities to or because of a greater focus on coursework, research, and teaching.

#### Negative and positive incentives

Perceptions of the potential rewards or negative outcomes from engagement can shape frequency of and views of engagement as well. These (dis)incentives can be intrinsic (e.g. a researcher enjoys engaging, on the positive side, or feels they are unable to effectively engage, on the negative side) or extrinsic (e.g. a researcher sees engagement as a way to increase citations rates, on the positive side, or hurting one’s scientific reputation, on the negative side) [12, 14, 41, 42].

In particular, we are interested in negative and positive incentives as they pertain to using social media for communication and engagement. In general, scientists in the U.S. appear to believe that communicating through social media can have positive advantages for their work [43] and for influencing public and policy-makers’ opinions [39]. Believing that lay audiences are interested (or not interested) in what scientists share can impact scientists’ perceptions of engagement overall [14, 36] and likely applies to engagement through social media as well. Additionally, some studies have found that scientists who share their articles through links on Twitter, for example, end up more heavily cited for those papers [44]. Scientists with more mentions on Twitter are more likely to have higher h-index scores, a measure of academic impact [45]. Research is mixed, however, on the extent to which using Twitter in particular to share scientific research actually does increase citations or advance scientists’ academic careers [46]. It is also unclear whether associations between Twitter mentions and academic impact are confounded by more newsworthy articles or more prominent researchers being more likely to be mentioned and cited, rather than mentions themselves leading to increased academic impact [44].

One study at a national research institute found that engaging through different media did not appear to harm researchers’ advancement in their careers over time compared to their colleagues who engaged less, and might have even have had a slight positive effect [15]. On the other hand, some researchers worry that sharing on social media could hurt their reputation as scientists, either from their colleagues or the public, which can be a disincentive for engagement outside of social media as well [16, 36]. Additionally, some perceive using social media as too time-consuming to use for work or engagement purposes [43].

Here we focus on extrinsic rewards, to have overlap between items included in the survey of graduate students in the biological and physical sciences and the survey of faculty in the same fields. The graduate student survey included items extrinsic and intrinsic rewards, while the faculty survey included only items on extrinsic rewards, partly because the survey focused on a larger set of issues than just engagement. As younger people are more likely to use and be familiar with social media than are older people, we are interested in the extent to which graduate students differently perceive (dis)incentives for using social media for engagement and sharing scientific research purposes. In other words, even if younger people are more likely to use social media in their everyday life, does that use transfer into different views of using social media in their academic careers and engagement as well?

#### Views of roles of scientists and the public

Additionally, we are interested in differences between graduate students and faculty scientists in how they conceive of outreach, the public, and their roles as scientists. The qualitative and quantitative research on these views typically finds that scientists have overall positive views of engagement and believe that it is part of their job to communicate their research and its implications to the public [11, 17, 38, 47]. The perceived necessity of communicating can often relate to views of engagement that are primarily tied to ideas of “defending science” or generating public support–and therefore continued policy and financial support–for science [11, 16, 18, 19, 38, 47] Scientists also frequently mention perceiving engagement as a way to recruit future scientists [16, 18].

The research described above suggests that the researchers surveyed envision engagement primarily as a one-way flow of information from scientists to the public. Qualitative studies capture this vision particularly well, and usually find that—corresponding to the one-way directional view of the purpose of engagement as providing information to the public–scientists still largely see the public as a group that is either ignorant or misinformed, with only a smaller portion of scientists seeing variety or capability among the public [18, 20, 47]. This view can be part of what makes engagement appear as risky, as it highlights the possibility of misunderstandings or a disconnect between the public and scientists that could result in losing public trust or support [18]. Some interviews with scientists have also captured a belief that although engagement can address public ignorance, there is limited room for increasing public interest in science [36]. This research did not address why they scientists believe that is the case, however–whether it is due to views of the public or to the scientists’ own concerns about personal ability to communicate well.

A minority of scientists in one study did express more complex views of the public and a belief in the opportunity for productive two-way discussion or debate [18]. The goal scientists expressed for that two-way discourse, however, was still that the public would become more like scientists, rather than public input shaping science or the scientists’ views [18]. Some research suggests, however, that public engagement is becoming more interactive (or two-directional), although the extent to which this is due to a few active researchers versus an overall culture shift is unknown [48].

With engagement through social media, it is possible that scientists’ views of the roles of the public and of scientists could change depending on the type of engagement and the nature of the discourse that social media facilitates. Two-directional engagement can be more easily accomplished on social media in some cases, through comments and discussion of scientific issues in real-time with interested publics on sites such as Twitter or sharing stories with friends through posts on sites like Facebook [9, 43]. Additionally, by providing scientists with a way to directly engage with the public without the mediating role of professional journalists, there is also the opportunity for different kinds of public conversations about science [10, 49, 50]. For example, scientists can now more easily publicly discuss the validity of particular studies, share studies before peer review, and comment on controversial issues in science and society.

Such conversations, however, although possibly facilitated by social media, also challenge previously held ideas that discussions on the validity of scientific results and on controversial science issues should occur only among scientific colleagues and not between scientists and the public [50]. If graduate students have different views of engagement and of using social media for engagement, it is possible that they would see less of a divide between conversations between scientists and conversations between scientists and the public. Because of the opportunity to use social media for different types of public conversations about science–ones that potentially include discussing controversial issues in science, unpublished results, and the validity of published results–we are interested in whether graduate students’ views of the acceptability of such communication differs from faculty’s views. Additionally, because of the potential for views on two-directional engagement with the public through social media to relate to perceptions of the role of the public in science, we are interested in graduate students’ and faculty’s views of the role of scientists and of the public in discussions and decision-making concerning science.

Altogether, this analysis provides a picture of views among graduate students and faculty research on using social media for public engagement and how these views relate to different beliefs on what kinds of scientific information scientists should publicly share and on the extent to which communication should be one- or two-directional between scientists and the public. Based on the literature on scientists’ engagement, we predict that:

H1a: Faculty engage with reporters more frequently than do graduate students.
H1b: Faculty engage with the public more frequently than do graduate students.
We are more interested, however, in addressing the following research questions:
RQ1: Do graduate students biological and physical sciences have a different view than do faculty in the same fields of the (dis)incentives of using social media for public engagement?
RQ2: What are the differences between graduate students and faculty in biological and physical sciences in their views of the acceptability of publicly discussing controversial scientific results, validity of results, and sharing results before peer review?
RQ3: Do graduate students biological and physical sciences have different views than do faculty in the same fields of the role of scientists and of the public in discussions concerning scientific research?

## Methods

### Data–faculty and graduate student surveys

All parts of data collection and analyses for this study were approved by the Institutional Review Board at the University of Wisconsin-Madison. Participants gave informed consent after receiving explanations of the study and of possible consequences. Prior to conducting the research, the authors underwent human participants research training for ethically conducting social science. The faculty survey was distributed to all faculty housed in biological and physical science departments on a large, Midwestern, land-grant university in May to June 2016 using the campus survey center. Respondents received an email with a link to take the survey online, and toward the end of the surveying window anyone who had not completed the survey received a letter with a paper copy of the survey. The online survey had 295 completes and the mail survey had 77, for a total for 372 completes (30 percent response rate).

The graduate survey was distributed to all enrolled graduate students through email, using a contact list from the university registrar’s office. Those who did not list an email contact were dropped (fewer than 10 percent), resulting in 8,264 students who received the survey. Responses were collected using the university’s Qualtrics Survey Hosting Service and fielded from January to February 2017 using multiple waves of email contact. The first contact was an emailed link to the survey with a brief summary of its purpose. A second email was sent to non-responders 1 week later, and a final email sent to any remaining non-responders 1 week after the second reminder. The completion rate was 11.3 percent, resulting in a final sample size of *N* = 931. To match the graduate student sample to the faculty sample, this analysis uses only those respondents who indicated they worked in physical or biological fields, leaving a sample of *N* = 480. Descriptives for each sample are listed in *Results*. A dataset with both the graduate student and faculty responses is available in Supporting Information (S1 File).

### Measures

Here we examine items capturing four areas scientists’ frequency and views of engagement:

1. Frequency of engagement with the public and with media
2. Views of rewards and barriers for using social media for engagement
3. Views of using social media for publicly discussing scientific results, especially for controversial science issues
4. Views of the public and public input.

Table 1 has the means and standard deviations for all the items.

**Table 1:** Mean comparisons (t-tests) of graduate students' and faculty members' frequency of engagement, views of rewards and types of appropriate discourse for engagement on social media, and views of the role of the public in engagement with science.

	Graduate students	Faculty
*How often do you engage in public outreach related to your research*?	*M* = 2.60 (*SD* = 0.96) N = 480	*M* = 3.15 (*SD* = 1.14) N = 371
	**T-statistic = 7.48 (p-value = <0.0001)**	
*How often do you talk to reporters about your research*?	*M* = 1.37 (*SD* = 0.68) N = 480	*M* = 2.42 (*SD* = 1.04) N = 370
	**T-statistic = 17.67 (p-value = <0.0001)**	
*Social media increases academic impact*, *such as citation rates*	*M* = 2.71 (*SD* = 0.87) N = 478	*M* = 2.71 (*SD* = 0.92) N = 370
	**T-statistic = 0.06 (p-value = 0.95)**	
*Social media is too time-consuming*	*M* = 3.16. (*SD* = 1.02) N = 479	*M* = 3.59 (*SD* = 0.98) N = 371
	**T-statistic = 6.13 (p-value = <0.0001)**	
*Social media negatively impacts my reputation as a scientist*	*M* = 2.46 (*SD* = 0.94) N = 479	*M* = 2.54 (*SD* = 0.83) N = 370
	**T-statistic = 1.24 (p-value = 0.21)**	
*Lay audiences interested in what I share about science on social media*	*M* = 3.49 (*SD* = 0.91) N = 479	*M* = 3.49 (*SD* = 0.86) N = 371
	**T-statistic = 0.04 (p-value = 0.96)**	
*Scientists should not discuss controversial topics on social media*	*M* = 1.58 (*SD* = 0.84) N = 479	*M* = 2.23 (*SD* = 0.90) N = 371
	**T-statistic = 10.82 (p-value = <0.0001)**	
*New findings should be communicated before peer review*	*M* = 1.92 (*SD* = 0.90) N = 479	*M* = 2.03 (*SD* = 0.94) N = 370
	**T-statistic = 1.69 (p-value = 0.09)**	
*Scientists should comment on the validity of published results on social media*	*M* = 3.15 (*SD* = 0.88) N = 479	*M* = 3.05 (*SD* = 0.84) N = 370
	**T-statistic = 1.62 (p-value = 0.10)**	
*Communicating does not affect public attitudes toward science*	*M* = 1.84 (*SD* = 0.82) N = 479	*M* = 1.88 (*SD* = 0.78) N = 370
	**T-statistic = 0.69 (p-value = 0.49)**	
*Public opinion is more important than scientists’ opinions when making decisions about ethical implications of scientific research*	*M* = 2.18 (*SD* = 0.92) N = 479	*M* = 2.24 (*SD* = 0.89) N = 370
	**T-statistic = 0.86 (p-value = 0.39)**	
*Scientists should pay attention to the wishes of the public*, *even when they think they're mistaken*	*M* = 3.33 (*SD* = 1.06) N = 479	*M* = 3.37 (*SD* = 1.07) N = 370
	**T-statistic = 0.46 (p-value = 0.64)**	
*Lay audiences can bring valuable perspectives to discussions about scientific research*	*M* = 3.81 (*SD* = 0.85) N = 479	*M* = 3.73 (*SD* = 0.86) N = 370
	**T-statistic = 1.36 (p-value = 0.17)**	

#### Frequency of engagement

We included two measures of engagement: one capturing frequency of engagement with the public and one capturing frequency of engagement with the media. The public engagement item asked respondents, “how often do you engage in public outreach efforts related to your field of research?” The media engagement item asked, “how often do you talk to reporters about your research?” Both items were measured on a 5-point scale: 1 = “Never”; 2 = “Less than once per year”; 3 = “A few times a year”; 4 = “Every few months to once a month”; 5 = “A few times a month.”

#### Perceived rewards and barriers for using social media for engagement

To capture a mixture of potential rewards and barriers that researchers perceive from using social media for engagement, we look at four items that ask respondents to indicate how much they agree that using social media or aspects of social media include particular (dis)incentives. The two items focused on rewards ask respondents how much they agree that:

1. “Using social media increases academic impact, such as citation rates.”
2. “There are lay audiences interested in what I have to say on social media.”

The two focused on barriers ask respondents how much they agree that:

1. “Using social media is too time-consuming.”
2. “Using social media negatively impacts my reputation as a scientist.”

All four items were measured on a 5-point scale from 1 = “Strongly disagree” to 5 = “Strongly agree.”

#### Views of discussing scientific results on social media

Because social media offer the opportunity to discuss findings in real-time and expand discussion of results beyond research circles, we are also interested in respondents’ views of using social media to publicly discuss or share information that might traditionally have been discussed only among scientists. These three items ask researchers how much they agree that,

1. “Scientists should not discuss controversial topics on social media.”
2. “New scientific findings of public interest should be communicated to the public, even before peer review.”
3. “After scientific findings are published, scientists should comment on their validity on social media.”

These items used the same 5-point scale from 1 = “Strongly disagree” to 5 = “Strongly agree.”

#### Views of the public and public input

Finally, we are interested in scientists’ views of the public and of what input the public can offer that is relevant to scientists’ work. We examine four items asking respondents how much they agree that:

1. “Communicating with the public does not affect public attitudes toward science.”
2. “Public opinion is more important than scientists’ opinions when making decisions about the ethical implications of scientific research.”
3. “Scientists should pay attention to the wishes of the public, even if they think citizens are mistaken or do not understand their work.”
4. “Lay audiences can bring valuable perspectives to discussions about scientific research.”

These items were also measured with the 5-point scale from 1 = “Strongly disagree” to 5 = “Strongly agree.”

To assess differences between graduate students’ and faculty members’ engagement experiences and views of social media and of the public, we ran t-tests comparing the differences in mean response in each group on each of the items described above.

## Results

Of the faculty sample, 30 percent of respondents are female, and the mean age is 51 years (SD = 11.51). Of the graduate students, 53 percent of respondents are female, and the mean age is 27 years (SD = 4.25). We do not have data to compare the demographics of each sample to the overall demographics of faculty and graduate students in the biological and physical sciences at the university of focus, nor do we have data on the average age of faculty and graduate students at the university. These samples are consistent, however, with faculty and graduate student gender breakdowns across all academic disciplines at the institution at the time of the surveys: of graduate students, 49 percent are female; of faculty, 32 percent are female.

Many similarities and several significant differences exist between the graduate students and faculty in biological and physical sciences in their frequency of engagement, views of the incentives of using social media for engagement, appropriateness of discussion topics on social media, and views of the public (Table 1).

### Frequency of engagement

**Fig 1:**
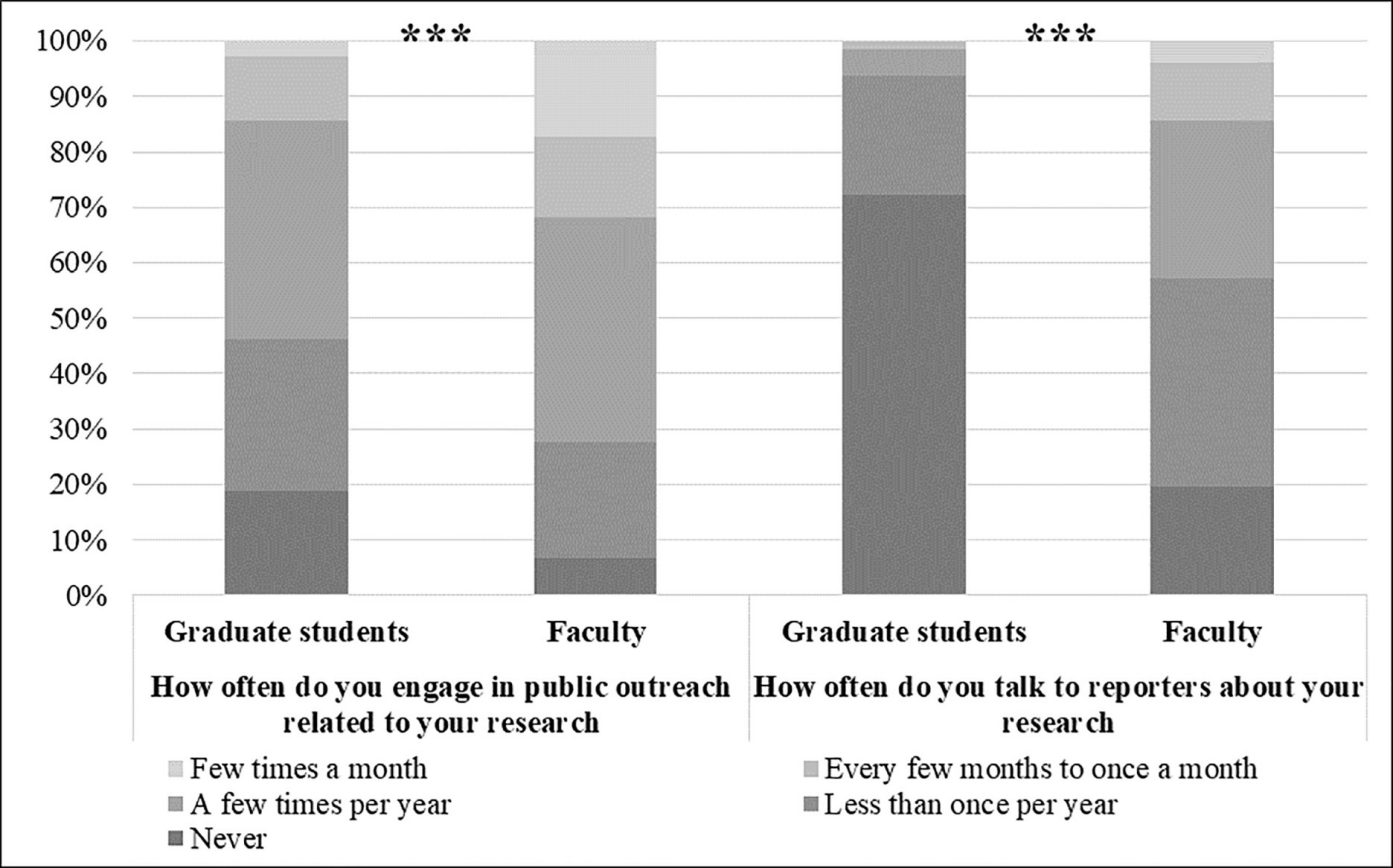
Frequency of engagement with the public and with reporters among graduate students and faculty members. Note: ***p≤0.001.

Faculty, perhaps unsurprisingly, engage with both the public and media significantly more often than do graduate students (Fig 1). With reporters, graduate students engage only very rarely on average (*M* = 1.37). Faculty, however, engage with reporters at least once or twice a year, or as often as graduate students engage with the public (*M* = 2.42), supporting H1a. With the public, graduate students on average engage slightly less than a few times a year, but still engage annually. Their mean of 2.61 falls between 2 on the scale, which indicates “Less than once per year,” and 3 which indicates “A few times a year.” Faculty, on average, engage with the public slightly more often, supporting H1b. They engage on average more than a few times a year (*M* = 3.15). Both groups engage with the public more frequently than they do with reporters.

### Perceived rewards and barriers for using social media for engagement

**Fig 2:**
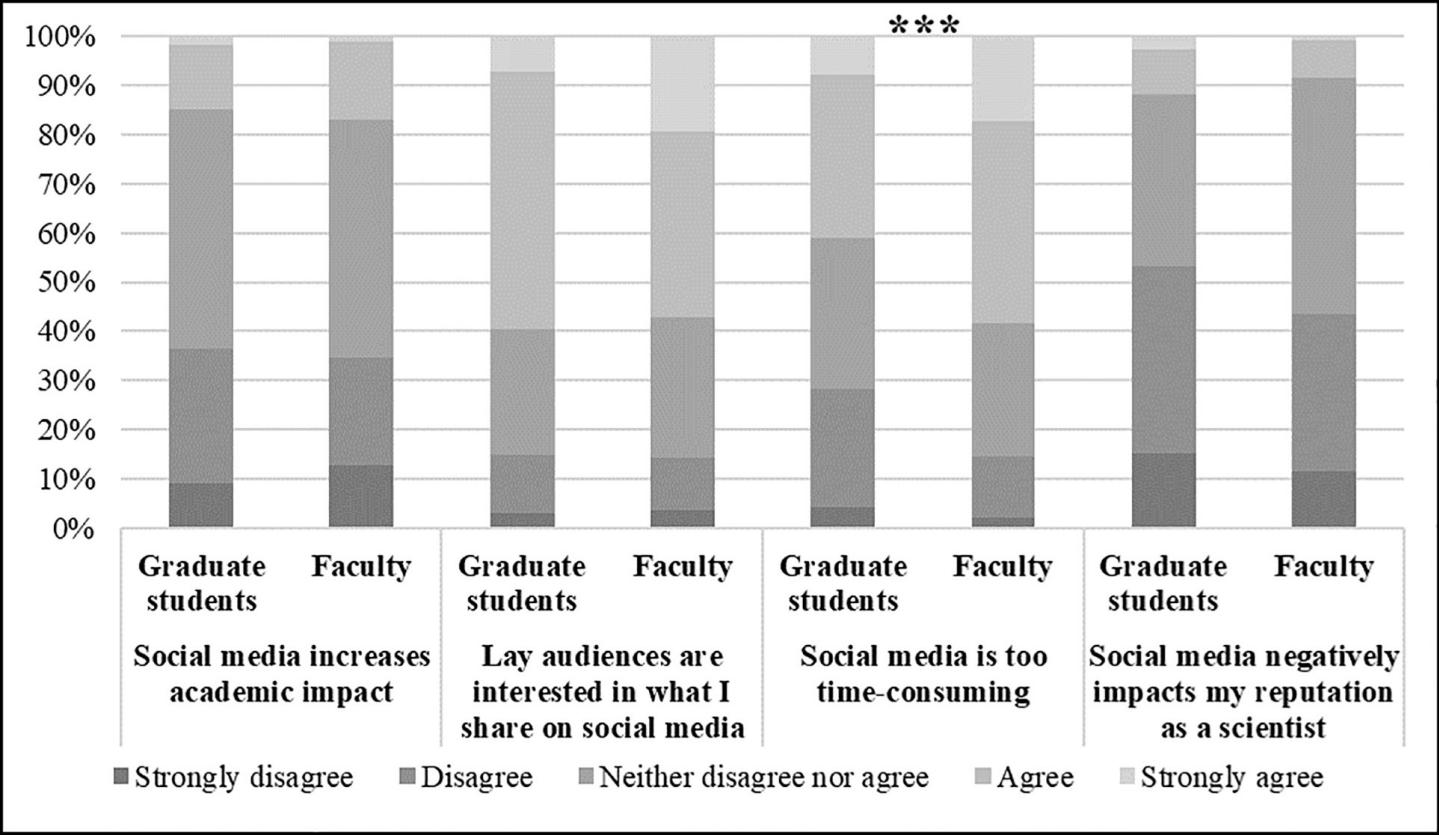
Percent agreement on perceptions of positive and negative incentives for engaging with the public on social media among graduate students and faculty members. Note: ***p≤0.001.

Overall, graduate students and faculty do not have very different perceptions of rewards or barriers to using social media, and when they do significantly differ it is in degree of agreement (Fig 2). Both groups fall between disagreeing or neither agreeing nor disagreeing that social media increases academic impact (graduate students: *M* = 2.71; faculty: *M* = 2.71) or negatively affects their reputations as scientists (graduate students: *M* = 2.46; faculty: *M* = 2.54). They also both lean toward agreeing that there are lay audiences interested in what they share on social media (graduate students: *M* = 3.49; faculty: *M* = 3.49). Graduate students and faculty do significantly differ, however, in their perceptions of social media as too time-consuming. Faculty on average lean toward agreeing that social media is too time-consuming (*M* = 3.59). Graduate students, on the other hand, lean more toward neither agreeing nor disagreeing (*M* = 3.16).

### Views of discussing scientific results on social media

**Fig 3:**
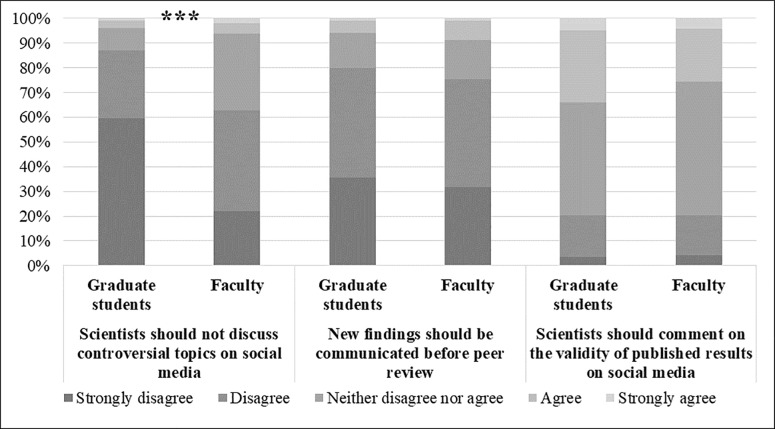
Percent agreement on perceptions of appropriate public discourse on social media among graduate students and faculty members. Note: ***p≤0.001.

Graduate students are more likely to find certain types of public discourse on social media more acceptable than do faculty. The direction of agreement, however, is mostly the same between the groups and they differ instead in intensity of agreement (Fig 3). Graduate students fall more toward strongly disagreeing that scientists should not discuss controversial topics on social media (*M* = 1.58). Faculty fall more toward disagreeing (*M* = 2.23). On whether scientists should comment on the validity of published results on social media, graduate students on average are closer to agreeing (*M* = 3.12) than are faculty, who are more solidly in the middle of the scale (*M* = 3.05), but the difference is not significant. Both groups are the same, however, in disagreeing that scientists should communicate new findings before peer review (*M* = 2.03 for both).

### Views of the public and public input

**Fig 4:**
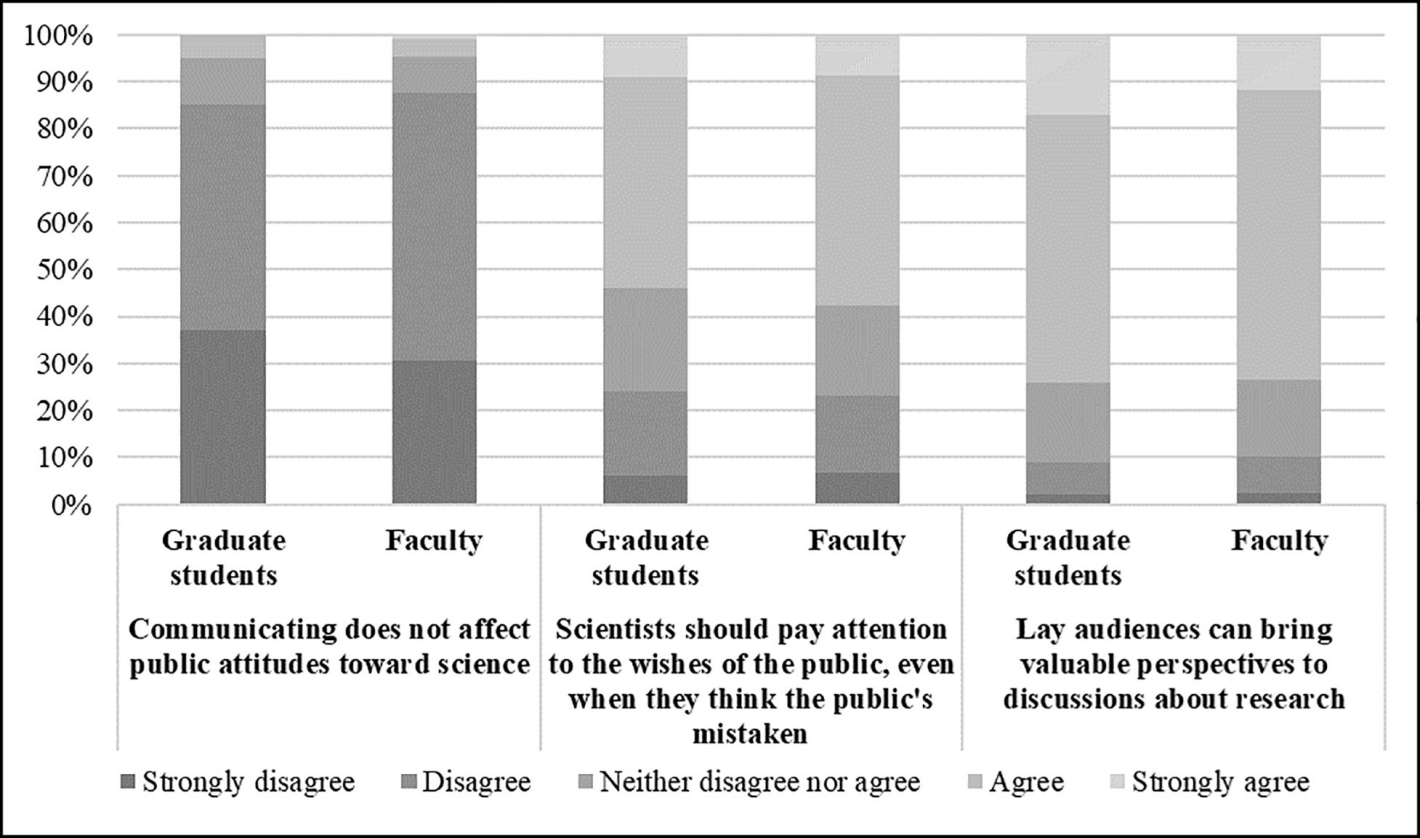
Percent agreement on perceptions of the public and of the role of scientists and the role of the public in engagement among graduate students and faculty members.

Finally, both groups overwhelmingly have positive perceptions of communicating with the public (Fig 4). Both agree that communication could affect public attitudes toward science (graduate students: *M* = 1.84; faculty: *M* = 1.88; reverse-wording item), and both agree that scientists should pay attention to the wishes of the public, even if they think that the public is mistaken (graduate students: *M* = 3.33; faculty: *M* = 3.37). Both also agree that that lay audiences can bring valuable perspectives to discussions about scientific research (graduate students: *M* = 3.81; faculty: *M* = 3.73). They also, however, both lean toward disagreeing that public opinion is more important than scientists’ opinion when making decisions about ethical implications of research (graduate students: *M* = 2.18; faculty: *M* = 2.24).

## Discussion

Although faculty in the biological and physical sciences at a large land-grant and research university in the United States engage with both the public and the media more often than do graduate students, the results provide some evidence that graduate students could view engagement differently than do faculty, especially through social media. Overall, however, we also see that both faculty and graduate students at the university of focus in this study have largely positive views of both social media and the public, and that when they do differ in their views it is largely in the strength of views (“strongly agree” versus “agree”) rather than holding opposite views of each other. We discuss the similarities and differences between the groups below.

### Shared views on the public and (dis)incentives for engaging through social media

First, focusing on the similarities between graduate students and the faculty, we see several positive outcomes regarding views of the public. Both faculty and graduate students believe that there are lay audiences interested in what they, as scientists, share on social media. Given the important role that perceptions of an interested public can play in encouraging engagement and in views of the purpose of engagement (e.g. convincing the public or defending science versus sharing ideas) [18–20, 47], this finding suggests that both graduate students and the faculty see social media as providing positive opportunities for engaging with the public. More generally, we also see that both groups overwhelmingly have positive perceptions of communicating with the public beyond social media. Both disagree that communication does not affect public attitudes toward science and both agree that the public offers valuable perspectives and that scientists should pay attention to the wishes of the public, even if they think the public is mistaken. These findings suggest that views concerning the potential ineffectiveness of communication are not a major barrier among either faculty and graduate students in this sample. Additionally, both groups largely see engaging as part of their duty as scientists and as a way to address public attitudes toward science.

Both groups also disagree that public opinion is more important than scientists’ opinion when making decisions about ethical implications of research. This finding, read in combination with both groups agreeing that scientists should pay attention to the public and that the public can provide valuable insights, suggests that the response that public views are not more important than scientists’ views might not reveal a negative view of the public. Instead, it could be interpreted as researchers not wanting to dismiss their own opinions about ethical implications of their work, rather than necessarily downplaying the role of the public.

Perhaps reflecting the mixed findings in other research on the effects on academic impact from sharing science information through social media [44–46], graduate students and faculty appear to be similarly mixed in their views. Both, on average, neither agree nor disagree that using social media increases academic impact or, on the flip side, that it harms their reputations as scientists. That both groups lean toward the middle on these items could suggest that they are not sure if using social media for engagement has these particular positive or negative effects. It could also reflect a “it depends” approach to viewing the potential incentives and disincentives of using social media for engagement. As poor communication can be worse than no communication, this more tempered view of the effects of engaging through social media–an environment in which scientists’ interactions can be shaped by context cues or easily spread beyond their control–is not necessarily a negative one. That researchers overall do not see a net negative effect of sharing through social media on their reputations as scientists is encouraging, as well, as fear of reputational damage, either among their peers or among lay publics, can be a disincentive to engaging [14, 51].

### Differences on views of the public and appropriate public discourse

Moving to the differences between graduate students’ and faculty members’ views, there are a few indicators of that younger researchers hold somewhat different views on engagement and information sharing with the public through social media.

First, graduate students are less likely to view using social media as time-consuming than are faculty. Given that many researchers cite time constraints as barriers to engaging [42], even with social media [43], this finding indicates that graduate students could be more likely to engage through social media. Further research, however, should more explicitly address the extent to which graduate students and faculty actually engage specifically through social media, rather than outreach in general, as the survey item we use here captured. Additionally, it would be helpful to see if this perception changes if graduate students become busier when they move on to faculty positions. Research on graduate student populations that compares different types of on- and off-line engagement contexts, similar to work conducted by Besley et al. [12] and Yuan et al. [11] on a sample of researchers with doctorate degrees, would also add to our understanding of this area. At the present, however, that graduate students do not view social media use as too time-consuming looks like an opportunity for engagement through those platforms.

Perhaps more interesting is that graduate students are more likely to find it acceptable to publicly discuss controversial topics on social media than are faculty. Graduate students, on average, strongly disagree that scientists should not discuss controversial topics on social media. The average faculty member also disagrees but without the same strength as the average graduate student respondent, and more faculty fall into the category of neither agreeing nor disagreeing. This finding could suggest that graduate students could have a different perception of what their roles are as scientists. As mentioned previously, traditional–although likely over-simplistic–perceptions of scientific norms draw lines between public discourse and discourse among scientific colleagues [50]. In that view, scientists protect professionalism by staying out of public discussions of controversial political or social issues and not publicly airing in-house disagreements about results [50].

The increasing prevalence of controversial and/or societally relevant issues that intersect with scientific research, findings, and technologies, however, could mean that more researchers either 1) become scientists to address these particular social and scientific issues or 2) are more likely, once engaged in scientific research, to see their fields as inseparable from societal issues and public support. In those cases, controversy could be part of what draws researchers to those fields, in the former example, or what they see as threatening the perceived legitimacy of and support for their research, in the latter. Younger researchers, then, could see publicly discussing controversy as a way of protecting and furthering their research and its purpose.

In future research, it would be interesting to build on these findings to better understand the cause and permanence of graduate students’ views of what types of science-focused discussion on social media are appropriate. That graduate students believe it is more acceptable to publicly discuss controversial issues could be interpreted as a change in what it means to protect and support science overall. With issues such as climate change, genetically-modified organisms in food, and vaccines, opponents to particular policy outcomes–such as reducing dependence on fossil fuels, relying on GMOs in the U.S. food system, or requiring vaccination in school children–can fuel controversy by questioning particular studies or citing studies that appear to challenge scientific consensus. A result of seeing such debates rage over the past few decades could be that younger researchers are more likely to believe in the importance of actively addressing such controversies.

Similarly, further examination of researchers’ views on commenting on the validity of published results on social media (which, in the samples here only 34% of graduate students and 25% of faculty agreed was appropriate, and pluralities of both groups neither agreed nor disagreed) would help us examine if the types of engagement that occur on social media are changing. Discussing validity of results, similar to addressing controversy, could represent scientists showing the public that they are policing science by publicly highlighting that science operates through checking and rechecking each result to strengthen validity. Such a view could also relate to recognizing a greater need for two-way engagement between scientists and members of the public [18, 24, 25, 30]. That graduate students are no different from faculty members in believing that scientists should not communicate new findings before peer review could reflect that graduate students appear to trust that peer review is a necessary part of the process of science and should operate before scientific information is shared.

### Limitations

Several limitations with the data in these analysis are important for interpreting and building on these results. First, the results represent one point in time at a university with a history of linking research to societal outcomes. As such, they could reflect the institutional culture of the university of focus in this study. As a land-grant and R01 institute, the university that both samples were drawn from combines a history of conducting science that reaches communities and members of the public with large research programs working on emerging, societally-relevant scientific research in the biological and information sciences–the fields that many see as requiring broader public discourse [10, 52]. At the same time, however, research finds that the combination of land-grant ideals with research-intensive priorities can create conflicting priorities and social cues that hinder engagement in practice even if institutional policies support engagement on paper [3]. With the scope of this study, we cannot know whether the differences between graduate students and faculty are larger or smaller than they would be at a different institutional setting.

Second, due to the cross-sectional nature of the data, we also cannot know the extent to which the findings indicate a lasting cohort shift in views of engagement versus just differences that will be socialized away as graduate students continue their training to become junior and senior faculty, as we discuss in greater detail below. Third, because not all graduate students and faculty who received the survey completed it, there is the potential for non-response bias. The students and faculty who responded to the survey might be more engaged or have different views of engagement and the public than does the larger pool of graduate students and faculty at this university. Finally, the items in the survey did not ask respondents to indicate what their definition of engagement was or how often they actually participate in engagement through social media or in particular types of discussions, such as around controversial issues and/or more one-way versus two-way communication media. More specific wordings and open-ended items would help us better connect the findings of graduate students’ and faculty’s views to specific engagement behaviors and parse out what type of public communication (one- or two-way) is more prominent among each cohort.

These limitations point to the need for research that examines other land-grant and non-land-grant universities, R01 and otherwise, and that follows graduate students overtime as they transition to faculty positions. Research that examines the extent to which graduate students and faculty use social media in particular ways as well will help connect attitudes toward social media, engagement, and the public to actual engagement behaviors and increase understanding of how social media is facilitating different types of engagement and discourse.

Research in other institutions can also provide us with a picture of how different institutional environments shape views and frequency of engagement. The samples here are from a university with a motto, well-known and supported across the campus, that signifies the university’s commitment to connecting research to public outreach and service. Because of this, both the graduate student and faculty groups could be particularly supportive of engagement and perhaps more aligned in their views than are researchers at institutions with less of a focus on such outreach. Whether there is indeed a shift to greater two-way engagement with science will also depend on the role of institutional culture and support. Institutional effects on graduate education training and curriculum incorporating engagement, rewards or disincentives for engagement among students and faculty, and socializing effects on what being a student, faculty member, and researcher looks like all play a role in facilitating or hindering engagement and future engaged scientists [1–3, 14, 17, 53]. Whether universities facilitate opportunities for interest in engagement to translate into actual engagement will likely affect whether engagement-inclined graduate students continue on to faculty positions at research institutions and if they are still engagement-inclined when they do.

## Conclusion

Graduate students appear to have slightly different views than do faculty members on how time consuming using social media is and of the acceptability of certain types of sharing of scientific information on social media. These differences suggest that there could be some meaningful differences in the way that younger scientists view their role as researchers and the types of engagement they participate in. They also highlight areas we should explore further to understand this understudied area of graduate students’ engagement and of the cultural and institutional factors that encourage or inhibit that engagement over a cohort’s academic career. The view that both faculty and graduate students hold of the public as contributing valuable insights lends itself to two-way engagement rather than the traditional tendency of scientists to picture or prefer one-way communication of information to lay audiences. For graduate students in particular, however, being less likely to view social media as too time-consuming could offer the opportunity for greater two-way engagement. Social media–as outlets for scientists to directly communicate with lay audiences in real time and in contexts that can combine scientific, societal, and political issues–appear to lend themselves to facilitating this kind of two-way engagement. The extent to which scientists use social media in this way remains to be studied.

That graduate students are more likely to perceive discussing controversial issues on social media as acceptable also suggests that graduate students might be more likely to perceive engagement as a two-way discussion with the public, and possibly that graduate students could hold a different view of their role as scientists. The fact that graduate students agree with faculty on the importance of allowing peer review to operate before sharing information from scientific results but believe more strongly in publicly discussing controversial science issues could provide insight into how graduate students view their role as scientists. It might be that such beliefs indicate that they see part of their role as protecting science by addressing the controversial issues aspects of scientific research today. In other words, graduate students might be more likely to believe they can protect the relevance and process of science overall by engaging in discourse on particular results and the implications of scientific research for societal issues and outcomes. Examining whether this is in fact the case and the extent to which these perceptions indicate a shift in researchers’ perceptions will require greater research into the views of graduate students and how their views relate to engagement practices and develop over time.

## Supporting information

S1 File IBM SPSS dataset file of graduate student and faculty responses (titled [dataset_grad student & faculty.sav]). (SAV)
